# Measuring (1,3)-β-D-glucan in tracheal aspirate, bronchoalveolar lavage fluid, and serum for detection of suspected *Candida* pneumonia in immunocompromised and critically ill patients: a prospective observational study

**DOI:** 10.1186/s12879-017-2364-2

**Published:** 2017-04-08

**Authors:** Kang-Cheng Su, Kun-Ta Chou, Yi-Han Hsiao, Ching-Min Tseng, Vincent Yi-Fong Su, Yu-Chin Lee, Diahn-Warng Perng, Yu Ru Kou

**Affiliations:** 1 Department of Chest Medicine, Taipei Veterans General Hospital, No.201, Sec. 2, Shipai Rd., Beitou Dist., Taipei City, 11217 Taiwan, Republic of China; 2 Center of Sleep Medicine, Taipei Veterans General Hospital, No.201, Sec. 2, Shipai Rd., Beitou Dist., Taipei City, 11217 Taiwan, Republic of China; 3 Institute of Physiology, School of Medicine, National Yang-Ming University, No.155, Sec.2, Linong St., Beitou Dist., Taipei City, 11221 Taiwan, Republic of China; 4 Division of Thoracic Medicine, Department of Medicine, Cheng Hsin General Hospital, No.45, Cheng Hsin St., Beitou Dist., Taipei City, 11220 Taiwan, Republic of China; 5 Sijhih Cathay General Hospital, No.2, Ln. 59, Jiancheng Rd., Xizhi Dist., New Taipei City, 22174 Taiwan, Republic of China; 6 School of Medicine, National Yang-Ming University, No.155, Sec.2, Linong St., Beitou Dist., Taipei City, 11221 Taiwan, Republic of China

**Keywords:** (1,3)-β-D-glucan, Bronchoalveolar lavage, *Candida* pneumonia, Critically ill patients, Diagnosis, Intensive care unit

## Abstract

**Background:**

While *Candida* pneumonia is life-threatening, biomarker measurements to early detect suspected *Candida* pneumonia are lacking. This study compared the diagnostic values of measuring levels of (1, 3)-β-D-glucan in endotracheal aspirate, bronchoalveolar lavage fluid, and serum to detect suspected *Candida* pneumonia in immunocompromised and critically ill patients.

**Methods:**

This prospective, observational study enrolled immunocompromised, critically ill, and ventilated patients with suspected fungal pneumonia in mixed intensive care units from November 2010 to October 2011. Patients with D-glucan confounding factors or other fungal infection were excluded. Endotracheal aspirate, bronchoalveolar lavage fluid and serum were collected from each patient to perform a fungal smear, culture, and D-glucan assay.

**Results:**

After screening 166 patients, 31 patients completed the study and were categorized into non-*Candida* pneumonia/non-candidemia (*n* = 18), suspected *Candida* pneumonia (*n* = 9), and non-*Candida* pneumonia/candidemia groups (*n* = 4). D-glucan levels in endotracheal aspirate or bronchoalveolar lavage were highest in suspected *Candida* pneumonia, while the serum D-glucan level was highest in non-*Candida* pneumonia/candidemia. In all patients, the D-glucan value in endotracheal aspirate was positively correlated with that in bronchoalveolar lavage fluid. For the detection of suspected *Candida* pneumonia, the predictive performance (sensitivity/specificity/D-glucan cutoff [pg/ml]) of D-glucan in endotracheal aspirate and bronchoalveolar lavage fluid was 67%/82%/120 and 89%/86%/130, respectively, accounting for areas under the receiver operating characteristic curve of 0.833 and 0.939 (both *P* < 0.05), respectively. Measuring serum D-glucan was of no diagnostic value (area under curve =0.510, *P* = 0.931) for the detection of suspected *Candida* pneumonia in the absence of concurrent candidemia.

**Conclusions:**

D-glucan levels in both endotracheal aspirate and bronchoalveolar lavage, but not in serum, provide good diagnostic values to detect suspected *Candida* pneumonia and to serve as potential biomarkers for early detection in this patient population.

**Electronic supplementary material:**

The online version of this article (doi:10.1186/s12879-017-2364-2) contains supplementary material, which is available to authorized users.

## Background

*Candida* pneumonia (CP) is life-threatening and has been associated with a high attributable mortality [1]. However, the definitive diagnosis is rarely established before overwhelming sepsis or death. Conventionally, a lung biopsy has been proposed to confirm CP [2, 3], but the procedure is too risky for patients in intensive care units (ICUs) because of the high prevalence of thrombocytopenia and coagulopathy [4]. Growth of respiratory *Candida* spp. in immunocompromised, cancer-afflicted, and critically ill patients is frequently found, but it is usually considered colonization rather than CP [5]. Indeed, growth of respiratory *Candida* spp. lacks specificity to diagnose CP [6–9], and adds little value to optimizing CP management [10, 11]. Recently, growing evidence has challenged the dogma that respiratory *Candida* spp. acts as a bystander in ICU patients [12]. The presence of respiratory *Candida* spp. has been shown to be associated with increased bacterial pneumonia development [13–16], selection of multidrug-resistant bacteria [17], and worse ICU outcomes [14, 18–20]. Accordingly, measurements of biomarkers with diagnostic value for the early detection of suspected CP are urgently needed.

Measurements of serum (1,3)-β-D-glucan (BDG), the common fungal wall antigen, can now be accomplished within hours. Measuring serum BDG has emerged as a rapid assessment to facilitate the diagnosis of invasive fungal infection, and it has proven to provide indirect mycological evidence for invasive fungal infection by a task force consensus [3]. A recent meta-analysis of 2979 patients included from 16 studies showed that using serum BDG levels to diagnose invasive fungal infection could attain a pooled sensitivity and specificity of 76.8 and 85.3%, respectively, and an area under the receiver operating characteristic curve (AUROC) of 0.89 [20]. However, none of these studies focused on respiratory *Candida* spp. infection. Additionally, it is conceivable that measuring BDG levels in respiratory specimens might be more accurate and specific for the diagnosis of pulmonary infection compared to measuring BDG levels in serum. Thus, the diagnostic value of measuring BDG levels in serum or respiratory specimens to detect suspected CP remains unclear.

In this study, we conducted a prospective, observational study aiming to compare the diagnostic value of measuring BDG levels in endotracheal aspirate (TA), bronchoalveolar lavage (BAL) fluid, and serum for the detection of suspected CP in immunocompromised and critically ill patients.

## Methods

### Study design

This prospective, observational study was conducted at medical and surgical ICUs in a medical center, Taipei Veterans General Hospital, in Taiwan, from November 2010 to October 2011. Eligible patients were recruited, and specimens of TA, BAL (see Additional file 1 for detail methods), and serum were collected from each individual patient on the enrollment day. BAL with or without transbronchial lung biopsy (TBLB) was a routine procedure at the discretion of the treating physicians in patients with unrecognized pulmonary lesions in our ICU. All the specimens were submitted to a bacterial culture, a fungal smear and culture, and a BDG assay. Fungal stain was applied routinely to inspect fungal structures microscopically. This study was approved by the institutional review board of Taipei Veterans General Hospital (IRB approval ID: 201011002IA) and signed informed consents were obtained from all participants.

### Patients

**Fig. 1:**
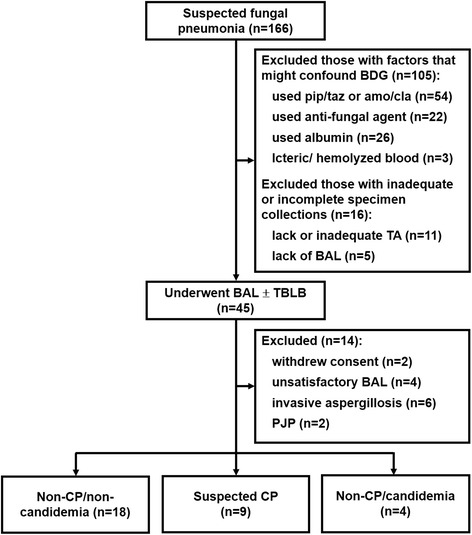
Study algorithm. pip/taz = piperacillin/tazobactam; amo/cla = amoxicillin-clavulanate; TA = endotracheal aspirate; BAL = bronchoalveolar lavage fluid; TBLB = transbronchial lung biopsy; PJP = *Pneumocystis jiroveci* pneumonia

Adult, mechanically ventilated ICU patients with suspected fungal pneumonia were recruited if they met all of the following conditions: presence of appropriate host factors defined by the task force consensus (see Additional file 1 for details about host factors) [3], non-responding or progressive pulmonary lesions despite management with broad-spectrum antibiotics for 48 to 72 h, and signature on an inform consent form. Patients were excluded if they withdrew consent, lacked respiratory specimens, had established diagnoses other than CP, or presented with factors that might confound the BDG assay (Fig. 1; see Additional file 1 for details about confounding factors) [10, 21–23]. The application of antifungal treatment depended on the treating team, which was blinded to the BDG results until ICU discharge. The final diagnosis was determined by at least 2 different ICU intensivists independently, including the primary physicians. A conference was held to determine the final diagnosis if a discrepancy existed.

### Diagnostic definitions

Suspected CP was defined if the cases met one of the 3 following conditions: 1) histopathological findings of polymorphonuclear leukocytes infiltration in lung, while the presence of hyphae and/or pseudohyphae in the bronchial lumen with or without peribronchial tissue invasion in TBLB specimens; 2) TA and/or BAL cultural yield of *Candida* spp. with successful antifungal treatment, which was indicated by improvement in attributable symptoms and signs, radiographic resolution of chest images, and clearance of C*andida* spp. in repeated specimens of TA (at least 2 times per week) or BAL (at the discretion of primary treating team); 3) TA and/or BAL-positive *Candida* spp. with antifungal treatment failure, which was characterized by persistent growth of *Candida* spp. in repeated TA or BAL specimens in conjunction with a lack of clinical and radiographic regression. For the later 2 conditions, there were no other reasonably causative factors (such as superinfection of viral or bacterial microorganisms) and the treatment response was modified from the guideline [24] recommended for invasive candidiasis other than CP. Non-CP indicated alternative diagnoses other than suspected CP, which included bacterial pneumonia, viral pneumonitis, pulmonary edema or a combination of these diseases, and all of these cases were discontinued from antifungal treatment within 7 days. Candidemia was defined if the blood culture was positive for *Candida* spp. growth. Based on these definitions, the final diagnoses were categorized into 3 groups: non-CP/non-candidemia, suspected CP, and non-CP/candidemia.

### Management of BDG assay

All the specimens for the BDG assay were collected and processed within 2 h of collection, followed by frozen storage at −70 °C until testing. The BDG assay was performed (see Additional file 1 for details about methods) using a Glucatell kit (Associates of Cape Cod, Inc., Falmouth, MA, USA) according to the manufacturer’s instructions. The results of the BDG assays were not used to categorize the final diagnosis.

### Statistical analysis

Data are presented as the mean ± SD, the median with inter-quartile range or a number (%), as appropriate. Continuous variables were evaluated using the Kruskal-Wallis test followed by the Mann-Whitney U test for pairwise comparisons. Categorical data were evaluated using the Chi-square or Fisher’s exact test. The association between two variables was evaluated using a Pearson’s correlation analysis. Analyses of ROC curves were performed to assess the predictive performance of BDG for suspected CP or candidemia in different specimens. The sensitivity, specificity, positive predictive value (PPV), and negative predictive value (NPV) of BDG levels for suspected CP were also calculated, and the best cutoff values were determined. *P* values <0.05 were considered significant for all tests.

## Results

### Patient characteristics

**Fig. 2:**
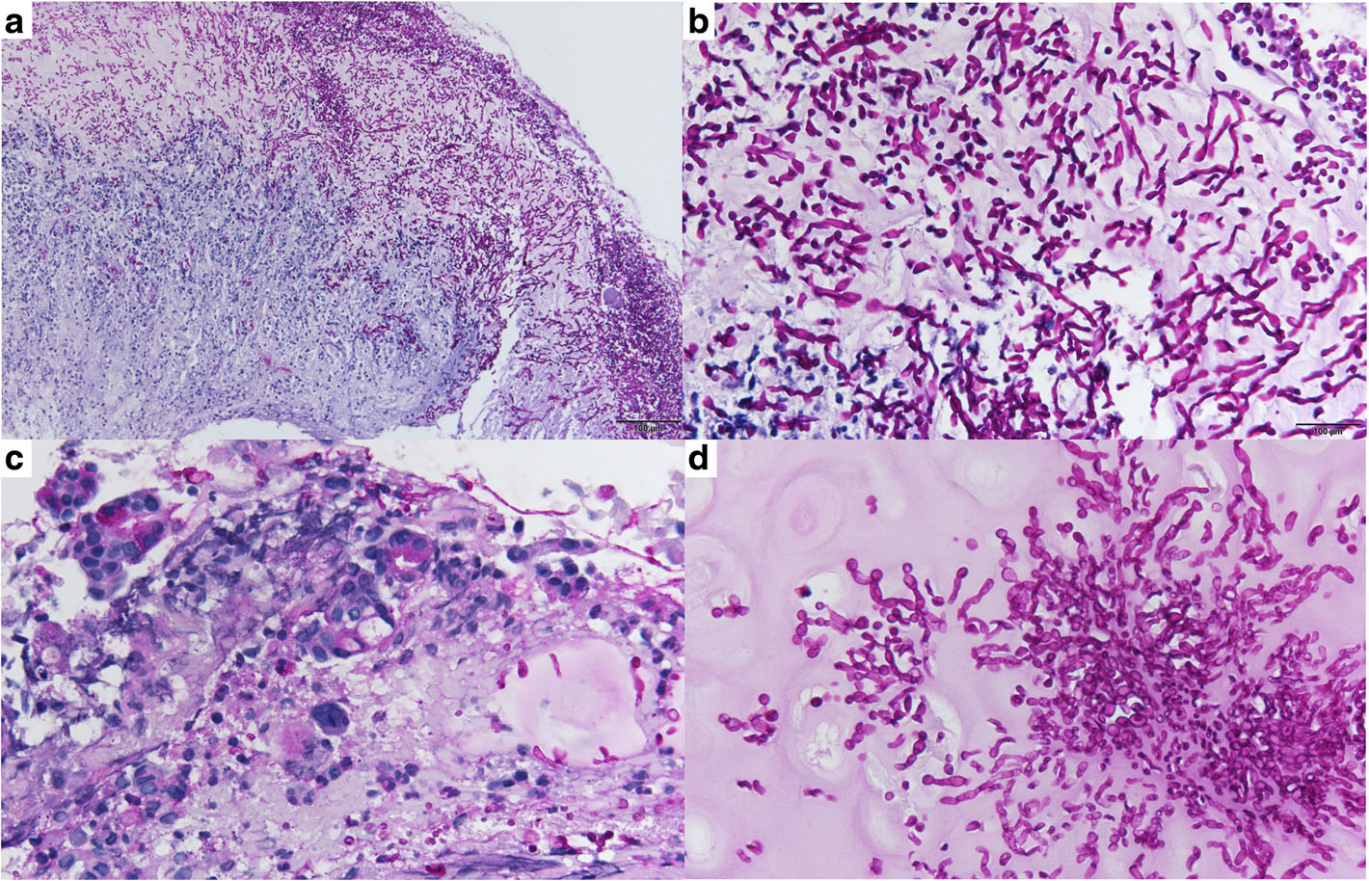
Histological findings of transbronchial lung biopsy with Periodic Acid-Schiff stain. A representative section of necrotic tissue admixed with inflammatory exudate showed infiltrations of acute inflammatory cells and fungus colonies in low magnification (**a**, 100X) as well as budding yeast and hyphae/pseudohyphae appearance in high magnification (**b**, 400X). Sections of peribronchial tissue disclosed epithelial sloughing with yeast invasion in bronchial mucosa (**c**, 400X) and bronchial cartilage (**d**, 400X)

After enrolling 166 consecutive patients with suspected fungal pneumonia, 31 patients completed the study. The final diagnoses were categorized as non-CP/non-candidemia (*n* = 18, 58.1%), suspected CP (*n* = 9, 29.0%), or non-CP/candidemia (*n* = 4, 12.9%) (Fig. 1). In suspected CP group, there were 4 patients with yeast in TBLB (including one with yeast invasion in peribronchial tissue, Fig. 2) and 5 patients judged by clinical response (3 failure and 2 success). Most of the patients’ characteristics were similar among the study groups (Table 1). The ICU mortality rate was 45.2% in total, but it did not significantly differ among the study group (Table 1). There were no independent factors to predict ICU mortality (please see Additional file 1: Table S1). However, all suspected CP cases demonstrated yeast evidence based on TBLB histopathology and/or BAL fungal staining, and they also had significantly higher *Candida* culture rates in BAL (Table 1). Details about the cultural results for the *Candida* spp. are shown in Additional file 1. All the patients were treated with empirical antifungal agents initially after specimen collection due to concern about immunocompromised status and high severity scores.

**Table 1:** Patients’ characteristics

	All	Non-CP/non- candidemia	Suspected CP	Non-CP/ candidemia	*P*
Number	31	18	9	4	
Age, year	66.4 ± 16.6	65.3 ± 18.5	66.9 ± 16.5	70.3 ± 6.2	0.971^a^
Sex, male (%)	20 (64.5)	11 (61.1)	6 (66.7)	3 (75.0)	0.860^b^
APACHE II at ICU admission	22.4 ± 5.2	22.3 ± 5.2	21.1 ± 4.2	25.3 ± 6.9	0.583^a^
APACHE II on enrolled day	24.2 ± 4.8	23.2 ± 5.0	25.1 ± 3.9	27.0 ± 5.3	0.316^a^
Initial reasons for MV (%)					
Post-surgery	7 (22.6%)	3 (16.7)	2 (22.2)	2 (50.0)	0.353^b^
Pneumonia	24 (77.4%)	15 (83.3)	7 (77.8)	2 (50.0)	0.353^b^
Reasons for BAL (%)					
Non-responding pneumonia	18 (58.1)	9 (50.0)	7 (77.8)	2 (50)	0.363^b^
Newly developed infiltration	13 (41.9)	9 (50.0)	2 (22.2)	2 (50)	0.363^b^
Host factors (%)					
Recent neutropenia	10 (32.3)	5 (27.8)	2 (22.2)	3 (75.0)	0.141^b^
Prolonged steroid use	16 (51.6)	10 (55.6)	5 (55.6)	1 (25.0)	0.521^b^
Immunosuppressants	5 (16.1)	3 (16.7)	2 (22.2)	0	0.600^b^
MV days	24.2 ± 12.5	23.7 ± 14.9	22.9 ± 9.4	29.3 ± 5.7	0.129^a^
ICU stay, days	27.8 ± 11.9	28.0 ± 14.4	26.6 ± 9.2	30.0 ± 4.3	0.494^a^
ICU mortality (%)	14 (45.2)	6 (33.3)	5 (55.6)	3 (75.0)	0.241^b^
Radiographic manifestations^c^ (%)					
Halo sign	1 (3.2)	0	1 (11.1)	0	0.283^b^
Single/multiple nodules	11 (35.5)	3 (16.7)	7 (77.8)	1 (25.0)	0.007^b^
Cavitary lesion	6 (19.4)	3 (16.7)	3 (33.3)	0	0.338^b^
GGO/infiltration	29 (93.5)	17 (94.4)	8 (88.9)	4 (100)	0.732^b^
Mycological evidence of yeast (%)					
TBLB^d,e^	4 (12.9)	0	4 (44.5)	0	< 0.001^b^
BAL^e^	13 (41.9)	4 (22.2)	8 (88.9)	1 (25.0)	0.003^b^
TA fungal culture	14 (45.2)	7 (38.9)	6 (66.7)	1 (25.0)	0.269^b^
BAL fungal culture	13 (41.9)	5 (27.8)	8 (88.9)	0	0.002^b^

Data are presented with means ± standard deviations

*CP Candida* pneumonia, *APACHE* acute physiology and chronic health evaluation, *MV* mechanical ventilation, *BAL* bronchoalveolar lavage, *GGO* ground glass opacity, *TBLB* transbronchial lung biopsy, *TA* endotracheal aspirate, *BAL* bronchoalveolar lavage fluid

^a^Kruskal-Wallis test

^b^Fisher’s exact test

^c^Each single patient could have more than one type of radiographic manifestation

^d^A total of 19 patients underwent TBLB, including 11 in non-CP/non-candidemia, 7 in suspected CP, and 1 in non-CP/candidemia

^e^Fungal staining was applied using Grocott-Gomori methenamine silver stain in BAL and Periodic Acid-Schiff stain in TBLB

### BDG levels among the study groups with different diagnoses

**Fig. 3:**
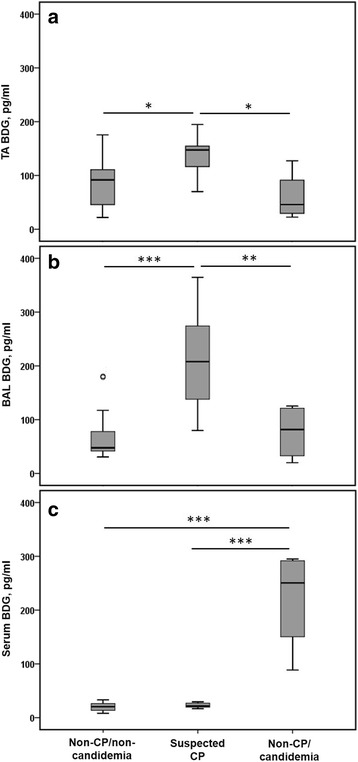
The levels of (1,3)-β-D-glucan (BDG) in specimens of endotracheal aspirate (**a**, TA), bronchoalveolar lavage fluid (**b**, BAL), and serum (**c**), categorized by different diagnoses. CP = *Candida* pneumonia. The shallow cycle indicates overlap of 2 outliers (180.8 and 178.0 pg/ml). *P < 0.05; **P < 0.01, ***P < 0.001. Statistical evaluations were performed using the Kruskal-Wallis test followed by the Mann-Whitney U test

The mean BDG level in TA (136.6 ± 37.1 pg/ml) was significant higher in patients with suspected CP than in those in non-CP/non-candidemia (85.7 ± 45.1 pg/ml, *P* = 0.022) and non-CP/candidemia (60.4 ± 46.6 pg/ml, *P* = 0.020) (Fig. 3a). The mean BDG level in BAL was also significantly higher in patients with suspected CP (217.5 ± 93.4 pg/ml) than those in the non-CP/non-candidemia (68.8 ± 46.5 pg/ml, *P* < 0.001) and non-CP/candidemia (77.1 ± 52.2 pg/ml, *P* = 0.003) groups (Fig. 3b). Conversely, the mean BDG level in serum in the non-CP/candidemia group (221.1 ± 96.0 pg/ml) was significantly higher than that in the non-CP/non-candidemia group (20.1 ± 7.5 pg/ml, *P* < 0.001) or the suspected CP group (22.7 ± 4.5 pg/ml, *P* < 0.001) (Fig. 3c).

### BDG level and Candida culture results

**Fig. 4:**
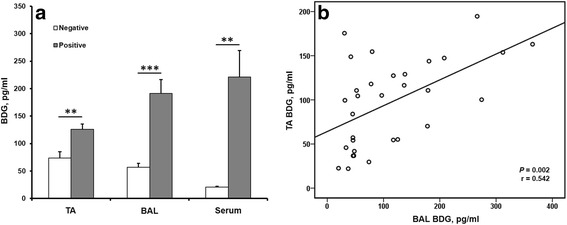
**a** BDG levels categorized by fungal cultural results in different types of specimens. **P < 0.01, ***P < 0.001. TA = endotracheal aspirate; BAL = bronchoalveolar lavage fluid. **b** Binary correlation of values of TA BDG and BAL BDG. r = correlation coefficient. Statistical evaluations were performed using the Mann-Whitney U test in A and using Pearson’s correlation coefficient in **b**

In all the patients, the BDG level was significantly higher in specimens with growth of *Candida* spp. than in those with a negative yield in each of the 3 types of specimens (126.2 ± 34.8 vs. 73.3 ± 47.9 pg/ml, *P* = 0.004 in TA; 191.0 ± 92.1 vs. 56.7 ± 30.2 pg/ml, *P* < 0.001 in BAL; 221.1 ± 96.0 vs. 21.0 ± 6.7 pg/ml, *P* = 0.001 in serum) (Fig. 4a). Additionally, the value of TA BDG was positively correlated with that of BAL BDG (*P* = 0.002, *r* = 0.542) (Fig. 4b). However, the value of neither TA BDG (*P* = 0.598; *r* = 0.098) nor BAL BDG (*P* = 0.836; *r* = 0.039) was significantly correlated with that of serum BDG.

### Diagnostic value of BDG in detecting suspected CP

**Fig. 5:**
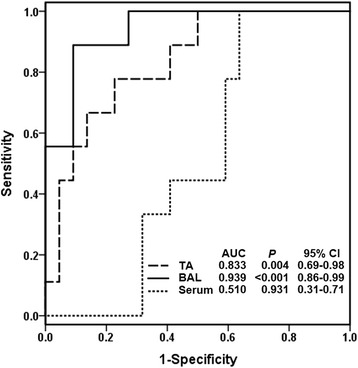
The receiver operating characteristic curve of BDG levels in different types of specimens for the diagnosis of suspected *Candida* pneumonia (CP). TA = endotracheal aspirate; BAL = bronchoalveolar lavage fluid; AUC = area under the curve; CI = conference interval

For the detection of suspected CP, measuring BDG levels in both TA and BAL, but not the serum BDG level, could have good diagnostic value. The results from the ROC analysis revealed that BDG levels in TA and BAL had AUROC values of 0.833 and 0.939, respectively (Fig. 5). Given a cutoff BDG level of 120 pg/ml in TA and 130 pg/ml in BAL, the best diagnostic value could be achieved with sensitivity/specificity of 67%/82% for TA and 89%/86% for BAL (Table 2). The serum BDG level exerted excellent predictive performance for the detection of candidemia (AUROC =0.996, *P* = 0.001) but poor predictive performance for the detection of suspected CP (AUROC =0.510, *P* = 0.931, Fig. 5). In addition, measuring the BDG level in TA (AUROC =0.259, *P* = 0.126) or BAL (AUROC =0.389, *P* = 0.480) for the detection of candidemia was of no diagnostic value.

**Table 2:** Predictive performance of various BDG levels for diagnosing candidemia and suspected CP in different specimens

Cutoff value	Sensitivity	Specificity	PPV	NPV
Candidemia				
Serum BDG, pg/ml				
70	100%	100%	100%	100%
80	100%	100%	100%	100%
90	75%	100%	100%	96%
Suspected CP				
TA BDG, pg/ml^a^				
90	89%	55%	44%	92%
100	89%	59%	47%	93%
110	78%	68%	50%	88%
120	67%	82%	60%	86%
130	0%	70%	0%	83%
BAL BDG, pg/ml				
110	89%	73%	57%	94%
120	89%	77%	62%	94%
130	89%	86%	73%	95%
140	89%	91%	80%	95%
150	67%	91%	75%	95%
Serum BDG, pg/ml				
20	67%	41%	32%	75%
25	33%	59%	25%	68%
30	0%	73%	0%	64%

*PPV* positive predictive value, *NPV* negative predictive value, *BDG* β-D-glucan

Refer to Table 1 for other abbreviations

^a^Adjusted to per gram of endotracheal aspirate

## Discussion

To our knowledge, the present study was the first to compare the diagnostic value of measuring BDG levels in TA, BAL, and serum for the detection of suspected CP in immunocompromised and critically ill patients. The major findings were that measuring BDG levels in respiratory specimens, including both TA and BAL fluid, exerted good diagnostic value for the detection of suspected CP, particularly in the absence of concurrent candidemia. In contrast, measuring the serum BDG level had no diagnostic value for this detection.

There are limited data regarding measuring BAL BDG levels to diagnose fungal pneumonia. The performance of BAL BDG at different cutoff value to diagnose non-*Candida* fungal pneumonia varied widely, in terms of sensitivity (53-90%) and specificity (26-88%) in different patient populations [25–28]. Among these, the largest study (268 BALs) reported by Prattes et al., who compared BAL galactomannan and BDG to diagnose proven/probable invasive pulmonary aspergillosis. They concluded that despite similar sensitivity (> 90%) and NPV (> 90%) between galactomannan and BDG, BAL BDG was less convincing due to low specificity (< 50%). This might be ascribed to frequent respiratory colonization of *Candida* spp. [28], which potentially limited the diagnostic performance of BAL BDG for non-*Candida* fungal pneumonia. In contrast, we excluded most of the possible confounding factors and focused on *Candida* pathogens. Thus, our data showed good performance (both sensitivity and specificity >85%) by means of measuring BAL BDG. Moreover, consistent with the observation reported by Reischies et al. [29], our data also showed that *Candida* culture-positive specimens had higher BDG levels (Fig. 4a), which implicates that BDG levels reflect fungal burdens indirectly. Patients with higher fungal burdens are more susceptible to fungal infection. This potentially provide the rationale to measure respiratory BDG levels as good diagnostic aid to detect suspected CP.

We further showed that the TA BDG level was positively correlated with BAL BDG (Fig. 4b). Despite with inferior value to BAL BDG, measuring TA BDG also offered good diagnostic performance for the detection of suspected CP (Fig. 5). Thus, measuring TA BDG could serve as a simple, less-invasive, alternative diagnostic for suspected CP when BAL is unavailable. Previously, most cases of CP were considered to indicate lung involvement of disseminated candidiasis rather than primary CP [1, 5]. Based on this belief, patients with CP are supposed to have as high of a serum BDG level as those with candidemia. However, our data did not correspond to this notion. Patients with suspected CP had apparently low BDG in serum (vs. those with non-CP/candidemia) and high BDG in TA and BAL (Fig. 3). These findings suggested that serum BDG might have no diagnostic value in patients with suspected CP in the absence of concurrent candidemia. Thus, we suggest clinicians should directly measure BDG in respiratory specimens in patients with suspicion of CP.

To date, there have been no universally acceptable criteria to diagnose CP, and most diagnoses have depended on lung biopsies. CP is considered uncommon based on earlier lung autopsy reports [1, 6, 8], so the clinical practice guideline does not recommend using antifungal treatment based upon the *Candida* culture alone [3, 5, 30]. However, CP might possibly be under-diagnosed in critically ill patients because a lung biopsy is rarely performed. Indeed, even for immunocompetent ICU patients with *Candida*-positive respiratory specimens, 24.2% of physicians recommended antifungal treatment in an early questionnaire surveillance regarding CP [31] and 32.9% did so in a recent prospective study concerning ICU-acquired pneumonia [32]. These reports reflected the dissociation of managements between the guideline [3, 5, 30] and real-life ICU situations remain an unresolved issue. It is logically speculated that antifungal treatment are more frequently used for immunocompromised and critically ill ICU patients, particularly while these patients were undergoing non-responding or progressive pneumonia with the presence of respiratory *Candida* spp. and the absence of the other causative pathogens after thorough work-ups. Thus, applying antifungal treatment response in these situations might be reasonably therapeutic diagnostics for suspected CP and could compensate the gap between the guideline and clinical scenario. Accordingly, more practical diagnostic criteria for detecting suspected CP are urgently needed. Our findings showed measuring respiratory BDG levels might provide valuable information to enhance these diagnostic criteria.

There were some limitations of our study. First, our data were derived from selected patients to minimize confounding factors. Particularly, we excluded many patients with concurrent use of antibiotics. The concern that if concurrent use of antibiotics possibly confounded BDG assay remains a conflicting issue [33–40]. It needs a large-scale study to validate this issue. Second, the sample size of this study was small, and future investigations with larger sample sizes will be needed to optimize the best TA or BAL BDG cutoff values. Third, our results were derived from the study of *Candida* spp.; the application of measuring BDG levels in other fungal pathogens requires further validation.

## Conclusions

This study is not intended to replace lung biopsy for the diagnosis of CP, but try to use less invasive biomarker to detect suspected CP. Our results clearly showed that the BDG level in BAL or TA provided a good diagnostic value to detect suspected CP. Additionally, measuring serum BDG had no diagnostic value for detecting suspected CP in the absence of concurrent candidemia. These findings warrant a future large-scale investigation to validate the optimal cutoff value for BDG in respiratory specimens and to determine the timing to initiate antifungal treatment.

## Additional file

Additional file 1: Supplementary Materials. (DOCX 24 kb)

## Abbreviations

AUROC: Area under the receiver operating characteristic curve
BAL: Bronchoalveolar lavage
BDG: (1,3)-β-D-glucan
CP: *Candida* pneumonia
ICU: Intensive care unit
NPV: Negative predictive value
PPV: Positive predictive value
TA: Endotracheal aspirate
TBLB: Transbronchial lung biopsy
