# Supplementary material for: Measuring (1,3)-β-D-glucan in tracheal aspirate, bronchoalveolar lavage fluid, and serum for detection of suspected Candida pneumonia in immunocompromised and critically ill patients: a prospective observational study
Source: BMC Infect Dis. 2017 Apr 8;17:252. doi: 10.1186/s12879-017-2364-2 (PMC5385026; doi:10.1186/s12879-017-2364-2)
Supplement: Supplementary file 1 — Supplementary Materials. (DOCX 24 kb) [file 12879_2017_2364_MOESM1_ESM.docx]

**Supplementary materials**

**Measuring (1,3)-β-D-glucan in tracheal aspirate, bronchoalveolar lavage fluid, and serum for detection of suspected *Candida* pneumonia in immunocompromised and critically ill patients: a prospective observational study**

**Performance of bronchoalveolar lavage**

Bronchoalveolar lavage (BAL) was performed while ventilated patients were sedated with full monitoring of vital signs. A fiberoptic bronchoscope (Model BF20 or P20; Olympus, Tokyo, Japan) was inserted through the artificial airway, followed by clearance of airway secretions and withdrawal of the bronchoscope to clear a working channel using sterile saline flushing. Subsequently, the bronchoscope was re-inserted and wedged into the orifice of a lobar or segmental bronchus of the appropriate lobe or segment, based on the location of pulmonary lesions on thoracic imaging. A total of two to three 50-mL aliquots of sterile isotonic saline were installed sequentially and were immediately recovered into a sterile collection container that was kept on ice during transport. The retrieved BAL fluid was subjected to bacterial culture, fungal culture, and Papanicolaou, Riu’s, and Grocott-Gomori methenamine-silver staining. The supernatants of BAL after the cytospin procedure were frozen and stored at −70°C until analyzed. The presence of any tracheal or bronchial lesions was recorded by the bronchoscopist. Samples of blind transbronchial lung biopsy or direct bronchoscopic biopsy were obtained whenever feasible. Either very mucous BAL fluids or the lack of alveolar macrophages in the cytospin smear were considered unsatisfactory BAL, and the specimens were discarded.

**Processing of endotracheal aspirate**

The processing of endotracheal aspirate (TA) was managed as induced sputum, as described previously [1]. The opaque and dense portions of TA were selected to obtain sufficient mucus plugs, followed by weighing to minimize dilution effects that might have influenced the final results. The pick-up mucus plugs were processed with 0.1% dithiothreitol (DTT). DTT does not interfere with the BDG assay, based on the manufacturer’s data. The cytospin sediment of BAL fluid was stained using May-Grunwald-Giemsa stain, and 400 non-squamous cells were counted. A sample was considered adequate when the percentage of squamous cells was < 20%. The supernatant of TA from cytospin was aspirated and frozen at −70°C until measurement was conducted using a BDG assay.

**Host factors and BDG confounding factors**

The host factors included patients who exhibited one of any following situations: 1. recent history of neutropenia (< 500 neutrophils/mm^3^ for > 10 days) temporally related to the onset of fungal disease; 2. receipt of an allogeneic stem cell transplant; 3. prolonged use of corticosteroids (at a minimum dose of 0.3 mg/kg/day of prednisolone equivalent for > 3 weeks); and 4. receipt of immunosuppressants (such as cyclosporine, TNF-α inhibitor, monoclonal antibody, nucleoside analogues etc.) over the previous 90 days [2]. The BDG assay could be confounded in patients with albumin infusion before samples collection or in those undergoing hemolysis or receiving icteric/hemolyzed blood [3-6] and possibly be interfered in patients with concurrent use of piperacillin-tazobactam [3, 7-9], amoxicillin-clavulanate [9, 10], or prior use of anti-fungal agents [11-13].

**BDG assay**

We applied a Glucatell kit to measure BDG because the Fungitell kit is not available in Taiwan. According to technical support from Technical Services Representative of Associates of Cape Cod, Inc., Fungitell and Glucatell kits (both are produced by Associates of Cape Cod) are identical kits for measuring BDG. However, the Fungitell kit is a medical device used specifically as one of the tools for the diagnosis of invasive fungal infection. The Glucatell kit is for research purposes only. All the specimens were collected in sterile BDG-free vacuum tubes. BDG measurements were completed within 6 months after sample collection. All the frozen specimens were thawed only once. All the assays were performed in duplicate in a microtiter plate, and the mean was used as the final result. Specimens (5 μL) were pretreated for 10 min at 37°C with an alkaline reagent (20 mL; 0.125 M KOH/0.6 M KCl) to inactivate serine proteases, as well as inhibitors, in human serum and to enhance the reactivity to activated factor G, according to the manufacturer’s instructions. After addition of the BDG assay reagent, the microtiter plate was inserted into a ThermoMax plate reader (Molecular Devices Corporation, Sunnyvale, CA, USA) and pre-incubated to 37°C, and a kinetic assay was run using SoftMax Pro software (Molecular Devices). In cases in which the duplicate results were discordant, the specimens were retested. A maximum coefficient of variation of 20% was expected for replicates of specimens with BDG values between 60 and 500 pg/mL.

**Cultural results of *Candida* species**

All the pathogens in cases of candidemia were *Candida albicans*. Of 31 paired respiratory specimens, 14 (45.2%) yielded *Candida* species in TAs (12 *Candida albicans,* 1 *Candida glabrata,* and 1 *Candida krusei*) and 13 (41.9%) in BAL (11 *Candida albicans,* 1 *Candida glabrata,* and 1 *Candida krusei*).

**References**

1. Perng DW, Huang HY, Chen HM, Lee YC, Perng RP. Characteristics of airway inflammation and bronchodilator reversibility in COPD: a potential guide to treatment. Chest. 2004;126:375-81.

2. De Pauw B, Walsh TJ, Donnelly JP, Stevens DA, Edwards JE, Calandra T, et al. Revised definitions of invasive fungal disease from the European Organization for Research and Treatment of Cancer/Invasive Fungal Infections Cooperative Group and the National Institute of Allergy and Infectious Diseases Mycoses Study Group (EORTC/MSG) Consensus Group. Clin Infect Dis. 2008;46:1813-21.

3. Ostrosky-Zeichner L. Invasive mycoses: diagnostic challenges. Am J Med. 2012;125:S14-24.

4. Hsu JL, Ruoss SJ, Bower ND, Lin M, Holodniy M, Stevens DA. Diagnosing invasive fungal disease in critically ill patients. Crit Rev Microbiol. 2011;37:277-312.

5. Alexander BD, Smith PB, Davis RD, Perfect JR, Reller LB. The (1,3){beta}-D-glucan test as an aid to early diagnosis of invasive fungal infections following lung transplantation. J Clin Microbiol. 2010;48:4083-8.

6. Pickering JW, Sant HW, Bowles CA, Roberts WL, Woods GL. Evaluation of a (1->3)-beta-D-glucan assay for diagnosis of invasive fungal infections. J Clin Microbiol. 2005;43:5957-62.

7. Sulahian A, Touratier S, Ribaud P. False positive test for aspergillus antigenemia related to concomitant administration of piperacillin and tazobactam. N Engl J Med. 2003;349:2366-7.

8. Adam O, Auperin A, Wilquin F, Bourhis JH, Gachot B, Chachaty E. Treatment with piperacillin-tazobactam and false-positive Aspergillus galactomannan antigen test results for patients with hematological malignancies. Clin Infect Dis. 2004;38:917-20.

9. Marty FM, Lowry CM, Lempitski SJ, Kubiak DW, Finkelman MA, Baden LR. Reactivity of (1-->3)-beta-d-glucan assay with commonly used intravenous antimicrobials. Antimicrob Agents Chemother. 2006;50:3450-3.

10. Mennink-Kersten MA, Warris A, Verweij PE. 1,3-beta-D-glucan in patients receiving intravenous amoxicillin-clavulanic acid. N Engl J Med. 2006;354:2834-5.

11. Senn L, Robinson JO, Schmidt S, Knaup M, Asahi N, Satomura S, et al. 1,3-Beta-D-glucan antigenemia for early diagnosis of invasive fungal infections in neutropenic patients with acute leukemia. Clin Infect Dis. 2008;46:878-85.

12. Ellis M, Al-Ramadi B, Finkelman M, Hedstrom U, Kristensen J, Ali-Zadeh H, et al. Assessment of the clinical utility of serial beta-D-glucan concentrations in patients with persistent neutropenic fever. J Med Microbiol. 2008;57:287-95.

13. Pazos C, Ponton J, Del Palacio A. Contribution of (1->3)-beta-D-glucan chromogenic assay to diagnosis and therapeutic monitoring of invasive aspergillosis in neutropenic adult patients: a comparison with serial screening for circulating galactomannan. J Clin Microbiol. 2005;43:299-305.

| **Table S1** Factors categorized by ICU outcomes. | | | | | | | | |  |
| --- | --- | --- | --- | --- | --- | --- | --- | --- | --- |
|  | Mortality | Survival | *P*^a^ | Univariate | |  | Multivariate | | |
|  |  |  |  | OR (95% CI)^b^ | *P*^b^ |  | OR (95% CI) | *P*^c^ | |
| Number (%) | 14 (45.2) | 17 (54.8) |  |  |  |  |  |  | |
| Age, year | 73.6 ± 7.4 | 60.5 ± 19.7 | 0.036 | 1.08 (1.0-1.2) | .049 |  |  |  | |
| Gender, Male (%) | 11 (78.6) | 9 (52.9) | 0.138 | 0.31 (0.06-1.51) | .146 |  |  |  | |
| APACHE II at ICU admission | 23.9 ± 6.0 | 21.1 ± 4.1 | 0.246 | 1.13 (0.96-1.31) | .134 |  |  |  | |
| APACHE II on enrolled day | 28.1 ± 2.7 | 21.6 ± 3.7 | <0.001 | 3.24 (1.15-9.10) | .026 |  | 1.60 (0.89-27.75) | 0.068 | |
| ICU stay, days | 31.5 ± 15.9 | 24.8 ± 6.2 | 0.201 | 1.06 (0.98-1.16) | .161 |  |  |  | |
| MV days | 30.7 ± 15.9 | 18.8 ± 4.6 | 0.006 | 1.21 (1.04-1.40) | .016 |  |  |  | |
| Direct microscopic finding of yeast (%) | |  |  |  |  |  |  |  | |
| TBLB | 2 (14.3) | 2 (11.8) | 0.422 | 2.0 (0.21-18.7) | .543 |  |  |  | |
| BAL GMS | 7 (50.0) | 6 (35.3) | 0.409 | 1.80 (0.43-7.78) | .411 |  |  |  | |
| Cultural growth of *Candida* spp (%) |  |  |  |  |  |  |  |  | |
| TA | 6 (42.9) | 8 (47.1) | 0.815 | 0.84 (0.20-3.50) | .815 |  |  |  | |
| BAL | 7 (50.0) | 6 (35.3) | 0.409 | 1.83 (0.43-7.77) | .411 |  |  |  | |
| Serum | 3 (21.4) | 1 (5.9) | 0.199 | 4.36 (0.40-47.61) | .227 |  |  |  | |
| BDG, pg/ml |  |  |  |  |  |  |  |  | |
| TA^d^ | 101.1 ± 50.5 | 94.0 ± 50.1 | 0.681 | 1.01 (0.99-1.02) | .690 |  |  |  | |
| BAL | 158.0 ± 111.4 | 76.0 ± 50.3 | 0.048 | 1.13 (1.01-1.03) | .028 |  | 0.042(0.99-1.01) | 0.127 | |
| Serum | 58.4 ± 86.5 | 37.3 ± 65.1 | 0.891 | 1.02 (0.99-1.01) | .442 |  |  |  | |
| Data are presented with mean ± standard deviation  *OR* odds ratio, *CI* conference interval, *BDG* beta-D-glucan, *APACHE* acute physiology and chronic health evaluation, *MV* mechanical  ventilation, *ICU* intensive care unit, *BAL* bronchoalveolar lavage, *TBLB* transbronchial lung biopsy, *GMS* Grocott-Gomori methenamine silver stain, *TA* tracheal aspirate, *BAL* bronchoalveolar lavage fluid  ^a^Chi-square test for categorical data and Mann-Whitney U test for continuous data  ^b^Univariate binary logistic regression model. OR indicates variables in mortality related to those in survival  ^c^Multivariate binary logistic regression model | | | | | | | | | |
